# Correlation of population mortality of COVID-19 and testing coverage: a comparison among 36 OECD countries

**DOI:** 10.1017/S0950268820003076

**Published:** 2020-12-28

**Authors:** C. Wei, C. C. Lee, T. C. Hsu, W. T. Hsu, C. C. Chan, S. C. Chen, C. J. Chen

**Affiliations:** 1 Harvard Medical School, Boston, USA; 2 Department of Emergency Medicine, National Taiwan University Hospital, Taipei, Taiwan; 3 Center of Intelligent Healthcare, National Taiwan University Hospital, Taipei, Taiwan; 4 Health Data Science Research Group, National Taiwan University Hospital, Taipei, Taiwan; 5 Department of Epidemiology, Harvard TH Chan School of Public Health, Boston, USA; 6 Institute of Environmental and Occupational Health Sciences, College of Public Health, National Taiwan University, Taipei, Taiwan; 7 Genomics Research Center, Academia Sinica, Taipei, Taiwan

**Keywords:** COVID-19, SARS-CoV-2, testing coverage

## Abstract

Although testing is widely regarded as critical to fighting the COVID-19 pandemic, what measure and level of testing best reflects successful infection control remains unresolved. Our aim was to compare the sensitivity of two testing metrics – population testing number and testing coverage – to population mortality outcomes and identify a benchmark for testing adequacy. We aggregated publicly available data through 12 April on testing and outcomes related to COVID-19 across 36 OECD (Organization for Economic Development) countries and Taiwan. Spearman correlation coefficients were calculated between the aforementioned metrics and following outcome measures: deaths per 1 million people, case fatality rate and case proportion of critical illness. Fractional polynomials were used to generate scatter plots to model the relationship between the testing metrics and outcomes. We found that testing coverage, but not population testing number, was highly correlated with population mortality (*r*_s_ = −0.79, *P* = 5.975 × 10^−9^ *vs. r*_s_ = −0.3, *P* = 0.05) and case fatality rate (*r*_s_ = −0.67, *P* = 9.067 × 10^−6^ *vs. r*_s_ = −0.21, *P* = 0.20). A testing coverage threshold of 15–45 signified adequate testing: below 15, testing coverage was associated with exponentially increasing population mortality; above 45, increased testing did not yield significant incremental mortality benefit. Taken together, testing coverage was better than population testing number in explaining country performance and can serve as an early and sensitive indicator of testing adequacy and disease burden.

Since the first case of coronavirus disease 2019 (COVID-19) was diagnosed in late December 2019, more than 50 million cases and 1.2 million deaths have been confirmed worldwide [1, 2]. Without fully approved drugs or vaccines, aggressive testing – directed at the SARS-CoV-2 genome – coupled with early isolation of exposed and infected patients, have been most effective at containing the pandemic. Except for countries with small populations however – Iceland being a notable example – mass screening is difficult, though widely recommended [3, 4]. This arises from the logistical hurdles of coordinating testing and contact tracing, which increases in difficulty with larger populations, along with the operational hurdles of ensuring enough tests and analytics are readily available.

Unable to test entire populations, determining the level of testing adequate to curb transmission is critical to guiding public health interventions. In recent days, this issue has become even more paramount as the USA and Europe experience rising case numbers after loosening business and social restrictions, and countries begin to rollback re-opening plans, including re-implementing lockdown measures. Both population testing number (tests per million people) and testing coverage (tests per confirmed case) have been cited as appropriate in this regard. This study compared the association between these two metrics and various country-level COVID-19 mortality outcomes, with the goal of identifying the more sensitive predictor of population mortality and deriving a widely applicable benchmark for adequate testing.

To this end, we collected open data from 22 January to 12 April on COVID-19 testing and outcomes for the 36 OECD (Organization for Economic Cooperation and Development) countries and Taiwan. The Spearman rank correlation test was conducted to evaluate the monotonic relationship between population testing number or testing coverage and several outcome measures, including population mortality rate, case fatality rate and proportion of critical illness. Scatter plots were generated and fitted by fractional polynomials to model the curvilinear relationship between each testing metric and outcome. A free-knot spline model was used to determine the optimal turning point. The analysis was conducted in R (R Foundation for Statistical Computing, Vienna, Austria) and scatter plots were produced using the package MFP. Comparisons were considered statistically significant for a two-sided alpha <0.05. This study was considered IRB-exempt as it involved analysis of de-identified, publicly available datasets.

We found that population mortality and case fatality rates were highly correlated with testing coverage (Spearman correlation coefficient (*r*_s_) = −0.79 and −0.67; *P* = 5.975 × 10^−9^ and 9.067 × 10^−6^, respectively) (Fig. 1a and 1b). In contrast, the correlation between population testing number and population mortality (*r* = −0.3; *P* = 0.05) and case fatality rate (*r*_s_ = −0.21; *P* = 0.20) were both weak (Fig. 2a and 2b). The proportion of critical cases was moderately correlated with testing coverage and population testing number (*r*_s_ = −0.51 and −0.50; *P* = 0.0017 and 0.0019, respectively) (eFigs S1A and S1B).

**Fig. 1.**
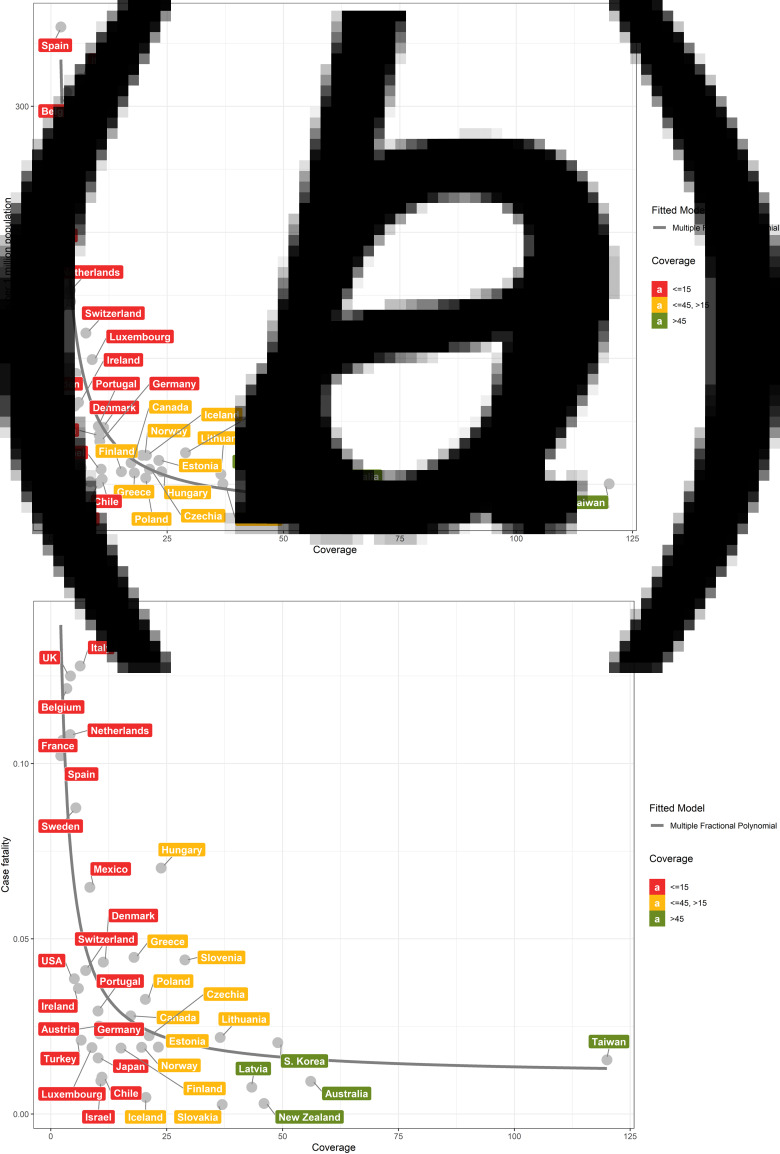
Scatter plots between coverage of tests and outcomes among the 36 OECD countries and Taiwan. The relationship between mortality (per 1 million people) of COVID-19 of 36 OECD countries and Taiwan and coverage of tests (a). The relationship between proportion of case fatalities and coverage of tests (b).

**Fig. 2.**
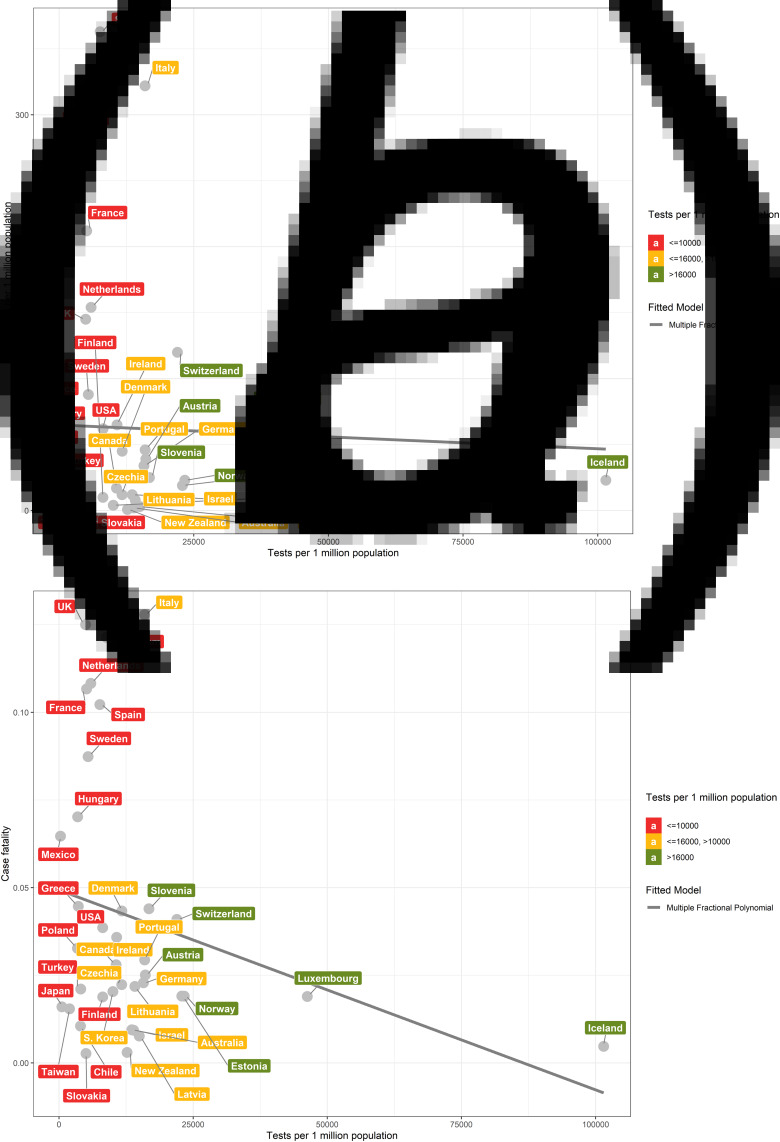
Scatter plots between population testing number and population mortality and case fatality among the 36 OECD countries and Taiwan. The relationship between mortality (per 1 million people) of COVID-19 of 36 OECD countries and Taiwan and number of tests per 1 million people (a). The relationship between COVID-19 case fatality and number of tests per 1 million people (b).

Detailed testing and outcome measures for each country through 12 April are summarised in Table 1. For the five countries with the lowest testing coverages, Spain (2.1), France (2.6), Belgium (3.4), Netherlands (4.2) and UK (4.2), the population mortality rates were 145, 154, 311, 212 and 363 per million people, respectively. In contrast, the five countries with the highest testing coverages, Taiwan (120.0), Australia (56.1), South Korea (49.0), New Zealand (46.0) and Latvia (43.3), reported population mortality rates of 0.3, 2, 4, 0.8 and 3 per million people, respectively.

**Table 1.** COVID-19 disease burden, outcome and testing number of 36 OECD countries and Taiwan

Countries	Confirmed cases	Population mortality (deaths/1 M)	Population testing number (tests/1 M)	Critical case ratio (%)	Test positivity rate (%)	Testing coverage
Taiwan	388	0.3	1954	NA	0.83	120.0
Australia	6313	2	13 880	2.8	1.78	56.1
South Korea	10 512	4	10 038	1.9	2.04	49.0
New Zealand	1330	0.8	12 684	0.6	2.17	46.0
Latvia	651	3	14 958	0.3	2.31	43.3
Slovakia	742	0.4	5023	0.7	2.71	37.0
Lithuania	1053	8	14 132	1.5	2.74	36.5
Slovenia	1205	25	16 764	3.5	3.46	28.9
Hungary	1410	10	3471	4.9	4.20	23.8
Estonia	1309	19	22 878	0.9	4.31	23.2
Czechia	5905	12	11 684	1.6	4.72	21.2
Iceland	1689	23	101 497	1.3	4.88	20.5
Poland	6356	5	3423	8.5	4.91	20.4
Norway	6459	23	23 332	1.1	5.11	19.6
Greece	2081	9	3583	4.4	5.57	17.9
Canada	23 318	17	10 639	3.4	5.81	17.2
Finland	2974	10	8125	3.1	6.61	15.1
Denmark	5996	45	11 700	2.8	8.85	11.3
Chile	6927	4	3995	7.7	9.07	11.0
Israel	10 878	12	13 557	1.9	9.27	10.8
Germany	125 452	34	15 730	7.5	9.52	10.5
Austria	13 945	39	16 086	3.7	9.63	10.4
Japan	6748	0.9	544	2.0	9.81	10.2
Portugal	15 987	46	15 966	1.5	9.82	10.2
Luxembourg	3270	99	46 272	1.1	11.29	8.9
Mexico	4219	2	275	4.1	11.89	8.4
Switzerland	25 300	120	21 954	3.2	13.32	7.5
Turkey	52 167	13	4036	3.4	15.33	6.5
Italy	152 271	322	15 935	3.4	15.80	6.3
Ireland	8928	65	10 734	2.3	16.85	5.9
Sweden	10 151	88	5416	8.9	18.56	5.4
USA	533 115	62	8138	2.4	19.79	5.1
UK	78 991	145	4934	2.3	23.58	4.2
Netherlands	24 413	154	5926	6.4	24.04	4.2
Belgium	29 647	311	8814	6.2	29.02	3.4
France	129 654	212	5114	7.7	38.84	2.6
Spain	166 019	363	7593	8.5	46.77	2.1

The inflection point of the mortality curve corresponded to a testing coverage of 15 and flattened after testing coverage exceeded 45 (Fig. 1a), and similarly for the case fatality curve (Fig. 1b). In contrast, there was only a mildly negative linear relationship between population testing number and population mortality and case fatality, respectively (Figure 2a and 2b).

Testing has become a paramount concern during the COVID-19 pandemic. However, few studies have evaluated how well different testing measures correspond to successful infection control and provide appropriate benchmarks to curb virus spread. Our investigation demonstrated that testing coverage, but not population testing number, was highly correlated with population mortality and case fatality, and that a threshold of 15–45 was adequate to minimise undetected cases and infection spread.

Being able to estimate the number of unmeasured cases in a population is critical to assessing testing adequacy. To this end, several groups proposed using a multiplier to convert confirmed case numbers into true case numbers using South Korea's and/or China's case fatality rate as a baseline [5, 6]. However, these methods can be confounded by country-specific biases in testing and reporting. Another approach relies on antibody testing. For example, one study found that infections in one US county were underreported by a factor of 54. However, this method is dependent on the accuracy of the underlying antibody assay and use in appropriate populations with high pre-test probability [7].

Our analysis provides an alternative approach using testing coverage, incorporating data from 37 countries rather than one–two countries. Assuming that the ability to detect the SARS-CoV-2 viral genome in testing samples has not changed over time and geography, our data suggest that countries with testing coverages of at least 45 need not increase testing, as further testing did not correspond to concomitant population mortality benefit, likely due to full capture of disease burden. Conversely, countries with testing coverages below 15 may need to ramp up testing and active surveillance.

In addition to informing ideal testing levels, testing coverage can estimate an area's true disease burden, calculated by multiplying the confirmed case count by the ratio of 15 to 45 and the area's current testing coverage. This can inform the degree of non-pharmacological intervention (NPI) needed to mitigate community transmission, which as ranked in eTable S1 can incur drastic societal and economic costs. Although countries such as the USA have been much maligned for their patchwork state-led response to COVID-19, to the extent that policy-making will continue to fall under state jurisdiction, testing coverage can be used as a guide at a local level to escalate or de-escalate NPIs in dynamic fashion [8]. In this regard, various seroprevalence tests evaluated by the FDA may provide further clarity from a population immunity standpoint [9]. It should be emphasised, however, that the experience of countries such as Taiwan and South Korea illustrate the value of centralised coordination of the pandemic response. Both countries quickly mobilised national response to ramp up comprehensive testing and contact tracing and used the test positive rate (one/testing coverage) to guide and evaluate the strategy of aggressive testing [10, 11]. In South Korea in particular, with increased testing, the cumulative positivity rate was 2.9% 19 days after the 100th case (compared to 17.4% in the USA) and continued to decline to 0.9% by 1 April [10]. For countries where testing is more limited and pandemic strategy – and individual compliance – varies by geography, as in the USA, testing coverage may be even more valuable.

The validity of testing coverage is dependent on several factors. Assuming that populations are not infected uniformly, testing accuracy may be subject to stochastic variation as well as systematic sampling bias. Therefore, its accuracy is dependent on access to testing, comprehensiveness of contact tracing and test sensitivity. Issues with these criteria that have arisen in various degrees include lagging testing infrastructure, contact tracing complicated by asymptomatic transmission and delayed discovery and low reverse transcription-polymerase chain reaction sensitivities ranging from 59% to 71% [12–15]. In spite of these limitations, our analysis showed that testing coverage was still highly correlated with country performance and carries additional benefits of low-cost and efficiency.

These results should also be interpreted in the context of other limitations. The negative correlation between testing coverage and population mortality does not imply causation, which can only be verified in a prospective interventional study – although there is anecdotal evidence suggesting that early antiviral treatment and/or supportive care may reduce mortality among COVID-19 patients [17], this may also be due to increased identification of patients with mild disease. In addition, the infection fatality rate of COVID-19 may vary from country-to-country, as has been seen in Italy [16]. Relevant modifiers include prevalence of risk factors, access to healthcare, robustness of healthcare infrastructure and population density. Indeed, generalising to non-OECD countries should be done with caution and further study should be conducted to verify this modality in these countries. Testing coverage should therefore be applied in context and with judgement rather than in a monolithic fashion.

In conclusion, we demonstrate the negative curvilinear relationship between testing coverage and COVID-19 population mortality and case fatality rate. Testing coverage can be used as both an indicator of testing adequacy and potential unidentified disease burden and is most accurate in the context of high healthcare accessibility, comprehensive contact tracing and testing sensitivity.

## Data Availability

The data that support the findings of this study are openly available in Worldometer at https://www.worldometers.info/coronavirus/.

## Supplementary material

For supplementary material accompanying this paper visit https://doi.org/10.1017/S0950268820003076. click here to view supplementary material
