# Supplementary material for: Correlation of population mortality of COVID-19 and testing coverage: a comparison among 36 OECD countries
Source: Epidemiol Infect. 2020 Dec 28;149:e1. doi: 10.1017/S0950268820003076 (PMC7804083; doi:10.1017/S0950268820003076)
Supplement: Supplementary file 1 [file S0950268820003076sup001.pdf]

Appendix

eFigure 1. Scatter plots between two testing metrics and proportion of critical cases among the 36 OECD countries and Taiwan. The relationship between proportion of critical cases and coverage **(A)**. The relationship between proportion of critical cases and number of tests per 1 million people **(B)**

eFigure 1A.

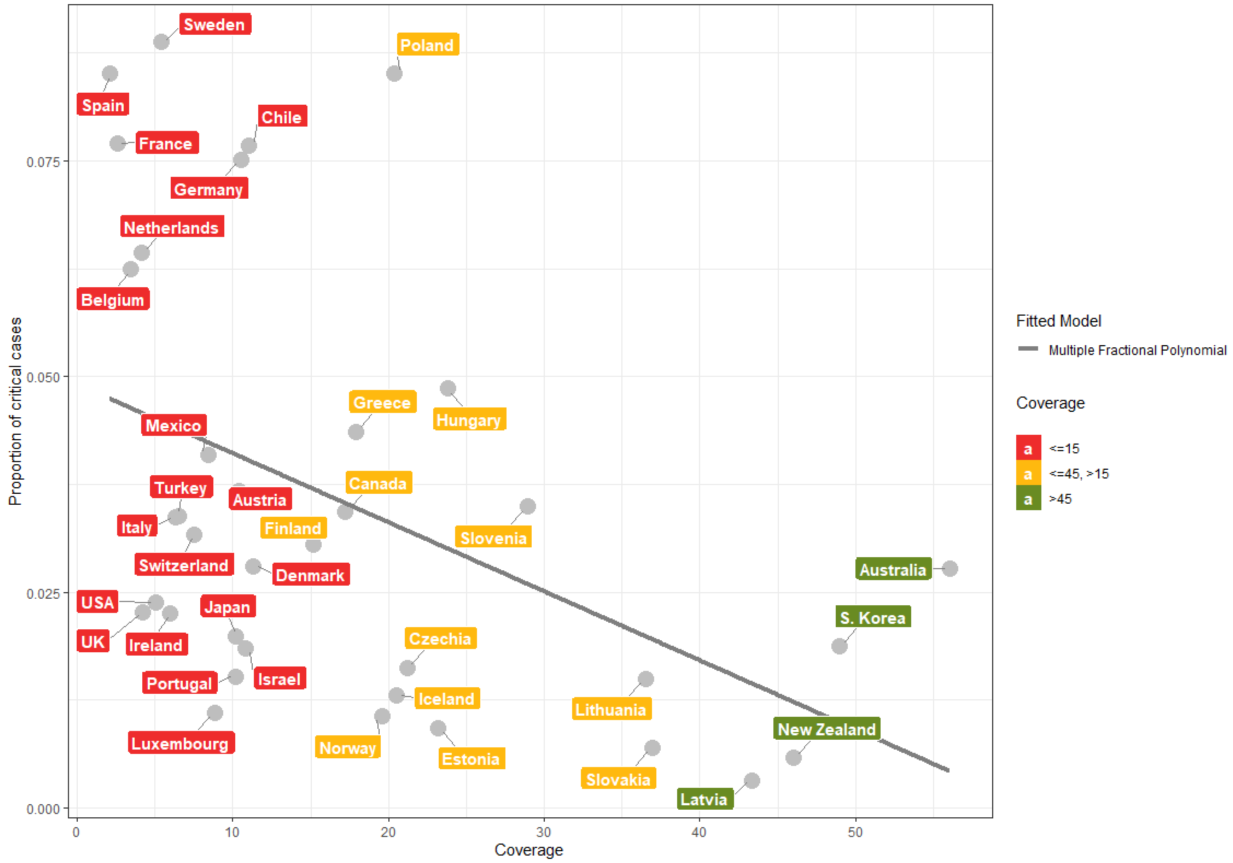

Formula: $y=-0.07998*x^{-1}+0.0491295$
Spearman correlation: -0.51
P-value: 0.001659

eFigure 1B.

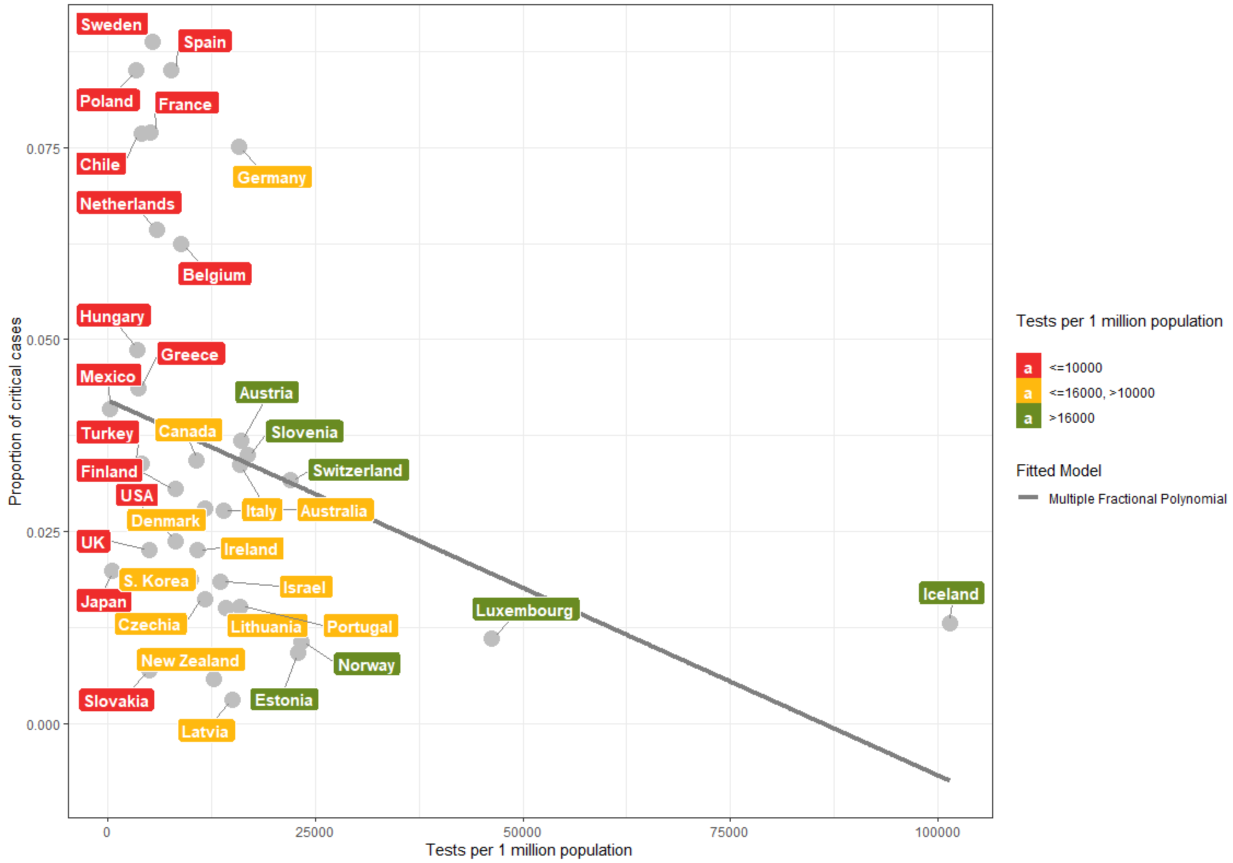

Formula: $y=-0.000488*x^{-1}+0.0421$
Spearman correlation: -0.50
P-value: 0.001883

eTable 1: Categorization of non-pharmacologic interventions to prevent COVID-19 spread

Level of Non-Pharmacological Intervention	Intervention Measures
1	Case isolation, home quarantine for suspected cases or exposed individuals, and wearing masks
2	Population-based social distancing such as closing schools, stadiums, churches and other public venues
3	Lockdown of an endemic area or an entire city.
